# Humoral immunoresponse elicited against an adenoviral-based SARS-CoV-2 coronavirus vaccine in elderly patients

**DOI:** 10.18632/aging.204299

**Published:** 2022-09-21

**Authors:** Rodrigo Hernán Tomas-Grau, Carolina Maldonado-Galdeano, Mónica Aguilar López, Esteban Vera Pingitore, Patricia Aznar, María Elena Alcorta, Eva María del Mar Vélez, Agustín Stagnetto, Silvana Estefanía Soliz-Santander, César Luís Ávila, Sergio Benjamín Socias, Dardo Costas, Rossana Elena Chahla, Gabriela Perdigón, Rosana Nieves Chehín, Diego Ploper, Silvia Inés Cazorla

**Affiliations:** 1 Instituto de Investigación en Medicina Molecular y Celular Aplicada - IMMCA (UNT-CONICET-SIPROSA), Tucumán 4000, Argentina; 2 Centro de Referencia para Lactobacilos – CERELA (CONICET), Tucumán 4000, Argentina; 3 Public Healthcare Administration (SIPROSA), Tucumán 4000, Argentina; 4 Néstor Kirchner Hospital, Central Public Health laboratory (LSP) (SIPROSA), Tucumán 4000, Argentina

**Keywords:** SARS-CoV-2, COVID-19, Gam-COVID-Vac, humoral immune response, elderly

## Abstract

The early sequencing of the SARS-CoV-2 viral genome allowed for a speedy development of effective vaccines against the virus. Nevertheless, age-related immunosenescence, the inability to mount strong immune responses, still represents a major obstacle. Here, in a group of 149 elderly volunteers (70–96 years old), evolution of the humoral immune response over time to Gam-COVID-Vac (Sputnik V), a vaccine based on heterologous recombinant adenovirus-26 (Ad26) and adenovirus-5 (Ad5) carrying the Spike genome, was analyzed by an anti-RBD ELISA. At 28 days post vaccination (dpv), a seroconversion rate of 91% was achieved, showing the importance of administering at least two doses of Gam-COVID-Vac to elicit a robust immune response, especially in elderly individuals without previous SARS-CoV-2 infection. Interestingly, IgG specific antibodies that reached their highest titers around 28 dpv (median = 740), persisted without significant decrease after 60 dpv (median = 650). After 90 dpv, IgG titers began to drop, and at 180 dpv only 44.7% of the elderly individuals remained with detectable anti-RBD IgG antibodies. No significant differences were observed in specific humoral immune responses between genders at early times point. However, at 60 dpv anti-RBD titers were more persistent in elderly females, and only dropped at 90 dpv (*p* < 0.0001). As expected, the highest antibodies titers were elicited in the youngest subgroup (70–74 years). Our results show that Gam-COVID-Vac was able to deal with the ageing of the immune system, eliciting a robust immune response in an elderly cohort, which lasted approximately 90 dpv at high levels, and protected against COVID-19.

## INTRODUCTION

The severe acute respiratory syndrome coronavirus 2 (SARS-CoV-2) that causes COVID-19, first reported in Wuhan, China in late 2019, has rapidly spread worldwide, causing a global pandemic. As of March 18, 2022, the virus resulted in over 462 million laboratory confirmed cases and more than 6 million deaths in 215 countries [1]. To manage this scenario, most governments were forced to implement highly restrictive lockdowns. Federal interventions have caused substantial economic losses and critical social consequences, affecting individuals’ behavior, mental health and social security.

COVID-19 is highly heterogeneous, ranging from asymptomatic, mild, severe and deadly. Host factors, including age, sex, and comorbid conditions, are key determinants of disease severity and progression [2]. SARS-CoV-2 affects greatly the most elderly and frailest fractions of the population. A characteristic of the elderly is a normal intestinal inflammatory process named “inflammaging” [3]. This condition damages healthy organs owing to increased vascular permeability, vascular paralysis, and hypovolemic shock, which lead to severe COVID-19 outcomes, which are associated with age [4]. Even more serious, aging leads to profound changes in both adaptive and innate immunity, resulting in increased susceptibility to infections and development of chronic inflammation [2, 5]. Alterations in all compartments of the immune system, including T and B cells, natural killer (NK) cells, macrophages and dendritic cells (DCs) and synthesis of interleukins (ILs), have been associated with age [6–8]. Decrease in both B cell populations and antibody production lead to a reduced efficacy in vaccination responses. Cellular senescence also contributes to the maintenance of inflammaging, due to acquisition of a senescence-associated secretory phenotype (SASP) by immune cells, secreting pro-inflammatory cytokines [9]. Moreover, Lin and colleagues proposed a hypothesis for the role of immune and inflammatory factors in contributing to coagulation dysregulation in the pathogenesis of COVID-19 [10]. An unbalanced pro-inflammatory response may also influence angiotensin converting enzyme-2 (ACE2) expression and facilitate viral entry [11]. Currently, the immune hypothesis integrates immunopathology secondary to cytokine storm, inflammaging and immunosenescence as a complex immune mechanism that influences the outcome of SARS-CoV-2 infection.

In the absence of antiviral medication, vaccines are the only option available to delay and reduce viral spread among the population. All vaccines aim to expose the body to an antigen that won’t cause disease, but will trigger an immune response that can block or kill the virus if a person becomes infected. Vaccine development against SARS-CoV-2 was initiated when the genetic sequence of the virus became available in January 2020 and has then moved at unprecedented speeds. Currently more than 180 vaccines, which rely on different vectors and platforms, are at various stages of development [12, 13]. Many of these haven’t been used in a licensed vaccine before. Gam-COVID-Vac (Sputnik V), uses a heterologous recombinant adenovirus 26 (Ad26) and adenovirus 5 (Ad5) as vectors that deliver the genetic sequence of the SARS-CoV-2 Spike protein, has been administered to tens of millions of volunteers worldwide, and has a good tolerability profile [14, 15].

Observational studies have attempted to assess the effects of the vaccines on particular variants of SARS-CoV-2, the persistence of vaccine protection, or both, when administered to different age groups [16]. The development of an effective vaccine against SARS-CoV-2 targeted for an elder population is a challenge [17]. Furthermore, there is limited data describing the behavior of COVID-19 vaccines when administered to the elderly. With this in mind, we analyzed the humoral immune response elicited against the Spike receptor binding domain (RBD) of SARS-CoV-2 in a prospective cohort study of 149 individuals, all 70–96 years-old, who received the complete scheme of the Gam-COVID-Vaccine, the only available vaccine in Argentine to the elderly population at the time of the study.

## RESULTS

### Gam-COVID-Vac elicited robust anti-RBD immune responses in elderly volunteers

In order to study the kinetics of immune responses to the COVID vaccine Gam-COVID-Vac in elderly individuals, 149 volunteers whose ages ranged from 70 to 96 years of age were enrolled. The basic demographics of the population studied are shown in Table 1. Humoral immune responses against the RBD of the SARS-CoV-2 Spike glycoprotein were evaluated at different time points, from 0 to 180 days post vaccination (dpv) by a previously published anti-RBD “*In House*” ELISA [18].

**Table 1:** Basic demographics of the elder volunteer population studied.

**Characteristics**	**Statistic**		
Number of study participants	*n*		149
Days Post Vaccination	*n* (%)	0	149 (100.0)
	*n* (%)	14	119 (79.9)
	*n* (%)	28	112 (75.2)
	*n* (%)	60	102 (68.5)
	*n* (%)	90	88 (59.1)
	*n* (%)	180	66 (44.3)
Sex	*n* (%)	Male	53 (35.3)
	*n* (%)	Female	96 (63.4)
	[M/F]	Sex Ratio	0.55
	*n*	No answer	0
Age-categories	Mean ± SD		76.1 ± 5.4
	*n* (%)	70–74 years	73 (49)
	*n* (%)	75–79 years	37 (24.8)
	*n* (%)	80–84 years	31 (20.8)
	*n* (%)	85–96 years	8 (5.4)
	*n*	No answer	0 (0)
Previous SARS-CoV-2 detection (TR-PCR or Rapid Ag Test) and	*n* (%)	Yes	7 (4.7)
	*n* (%)	No	142 (95.3)
	*n* (%)	No answer	0 (0)
Infections post vaccination	*n* (%)		1 (0.65)
		Age	72
		Gender	Male
		Hospitalized	No

**Figure 1:**
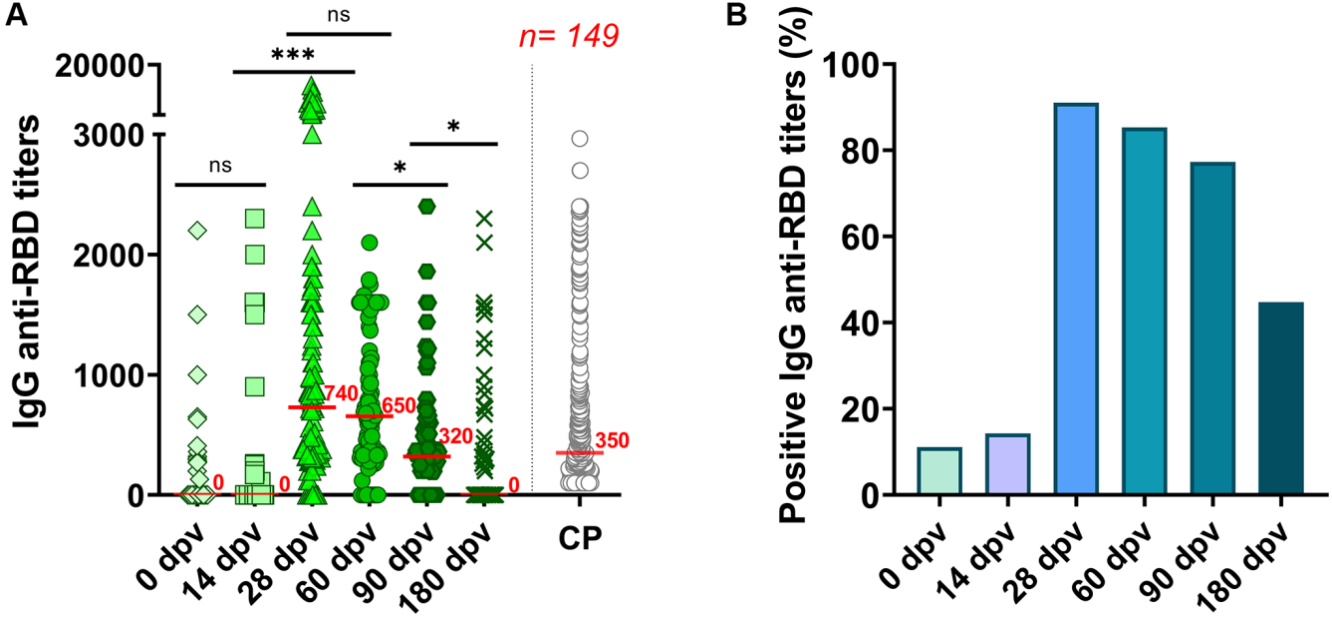
**Humoral immune response to the Gam-COVID-Vac in 149 elderly individuals.** (**A**) A cross-sectional study of IgG anti-RBD titers measured by ELISA in serum samples taken at 0, 14, 28, 60, 90 and 180 days’ post-vaccination (dpv). CP: SARS-CoV-2 Convalescent plasm (*n* = 309) analyzed with the same ELISA platform [17]. Statistical analyses were performed with Kruskal-Wallis test with two-stage linear step-up procedure of Benjamini, Krieger and Yekutieli. ^***^p < 0.0001; ^*^p < 0.05 and ns = not-significant. (**B**) Percentage of anti-RBD seropositive responses to the Gam-COVID-Vac vaccine in the population studied.

In a cross-section and prospective analysis of the 149 individuals studied, antibodies titers determined the day before vaccination with Gam-COVID-Vac administration revealed that nearly 11% of volunteers already presented antibodies against the RBD, suggesting a history of a previous SARS-CoV-2 infection (Figure 1A). Two weeks after vaccination with the first component of Gam-COVID-Vac (14 dpv), no statistical difference was observed between median antibodies levels detected compared to the basal time-point (*p* = 0.5541), and seroconversion increased only 3 percent, reaching 14.3% (Figure 1B).

At 28 dpv (7 days after administration of the second component), and having completed the vaccine scheme, high antibodies titers against RBD were detected (median titer = 740) (*p* < 0.0001 with respect to 0 dpv and 14 dpv). At this time point, the seroconversion rate raised up to 91% (Figure 1B).

We further analyzed the waning of anti-RBD responses to Gam-COVID-Vac at four subsequent time-points: 28, 60, 90 and 180 dpv. Although IgG specific antibodies reached their highest titers around 28 dpv (median titer = 740), they persisted without significant decrease after 60 dpv (median titer = 650) (Figure 1A). Nevertheless, the seroconversion rate decreased slightly to 85% (Figure 1B). After 90 dpv, median anti-RBD IgG titers began to decrease, reaching a median value of 320 (*p* = 0.0273 with respect to 60 dpv). For comparison, anti-RBD titers induced by viral infection with SARS-CoV-2 in convalescent individuals (CP), measured with the same ELISA platform for a different study [17] (see Materials and Methods) are also plotted (grey circles) (Figure 1A). As observed, elderly individuals vaccinated with Gam-COVID-Vac showed, after six months, similar anti-RBD titers as convalescent individuals one month after infection. In terms of seropositivity, six months after inoculation with the first dose (180 dpv), only 44.7% of the elderly individuals remained with detectable anti-RBD IgG antibodies (Figure 1A and 1B). The anti-RBD IgG antibody levels from 57 individuals that provided samples at all the time-points are shown in Supplementary Figure 1. This analysis showed nearly identical results as those obtained in the cross-section analysis of Figure 1A.

### Elderly individuals with previous SARS-CoV-2 infection elicited higher anti-RBD immune responses to Gam-COVID-Vac

**Figure 2:**
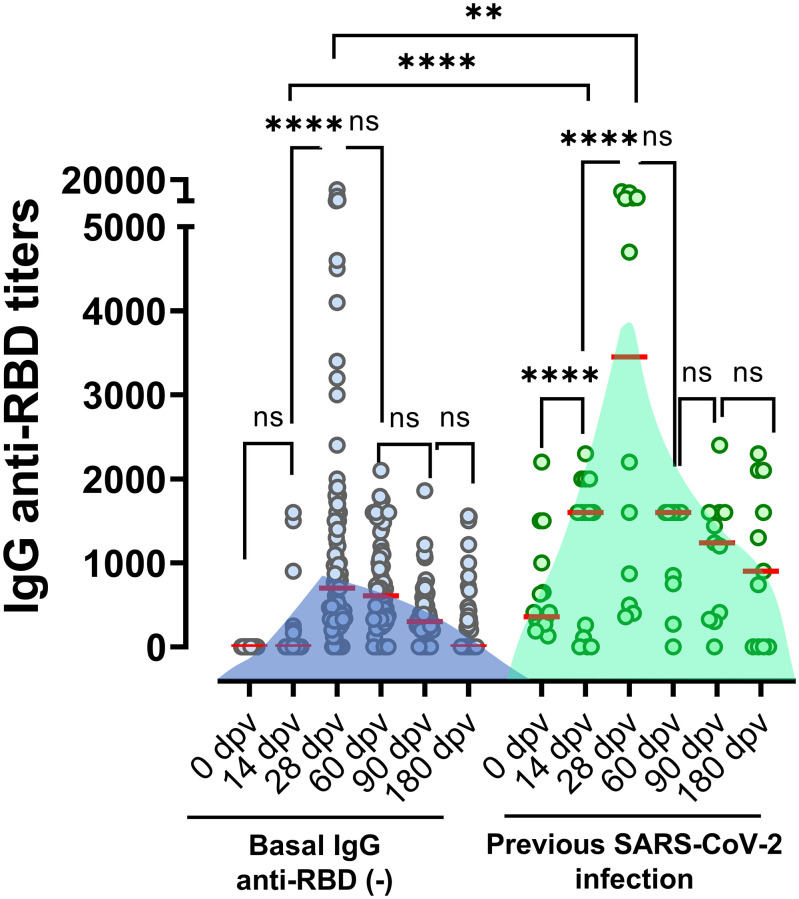
**Anti-RBD titers elicited in individuals with or without positives serology at baseline.** Antibodies titers in the studied population grouped according to documented absence or presence of previous SARS-CoV-2 infection (defined by positive RT-qPCR, rapid antigen test, or basal anti-RBD titer). Horizontal red lines represent the median. Statistical analyses were performed with Mann-Withney test.

Previous studies that analyzed different COVID vaccine platforms in younger individuals have shown that first doses may act as boosters in individuals previously exposed to SARS-CoV-2 [19]. Here we show that for elderly volunteers vaccinated with Gam-COVID-Vac this was also the case, as we observed that those individuals with detectable anti-RBD antibodies at baseline, developed higher antibody titers within 14 days of vaccination (Figure 2). By contrast, individuals without previous SARS-CoV-2 infection did not show an increase in antibody titers at 14 dpv. Nevertheless, one week after receiving the second component of Gam-COVID-Vac (28 dpv), a significant increase in antibody titers against the RBD was detected, independent of previous COVID-19 infection status (Figure 2). At 60 and 90 dpv specific antibodies were still high, regardless the previous infection status, while at 180 dpv only those with history of viral infection presented detectable anti-RBD IgG.

### Anti-RBD immune responses to Gam-COVID-Vac in elderly individuals as a function of gender and age

**Figure 3:**
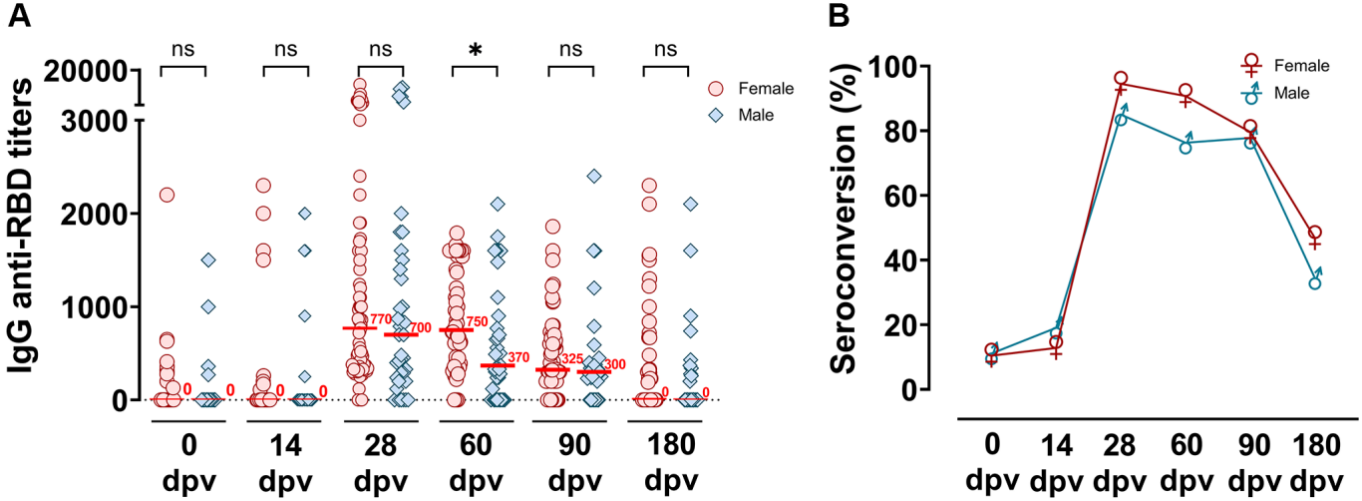
**Distribution of IgG anti-RBD titers measured up to 180 dpv differentiated by gender groups.** (**A**) Specific SARS-CoV-2 anti-RBD antibody titers were analyzed according to male or female gender groups, before and at different times post-vaccination (0, 14, 28, 60, 90 and 180 dpv) by ELISA. Horizontal red lines represent the median. Statistical analyses were performed with Mann-Withney test. ^*^p < 0.05. (**B**) Seroconversion of IgG anti-RBD among the time post-vaccination in female and male groups.

**Figure 4:**
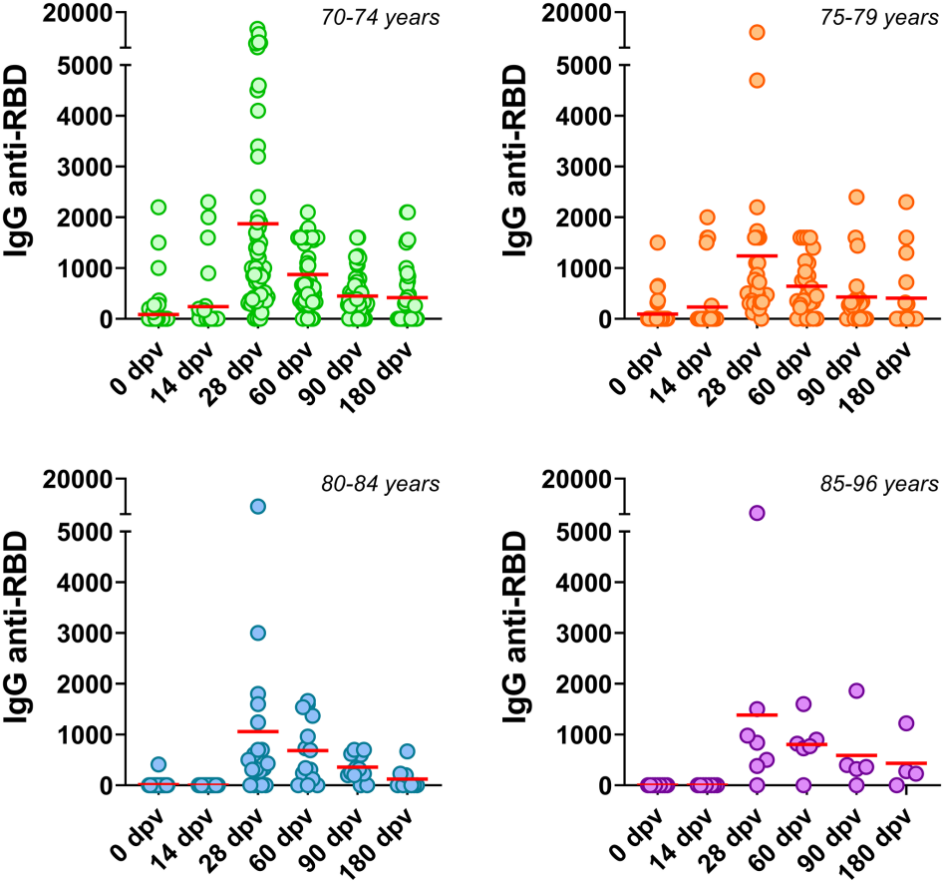
**Anti-SARS-CoV-2 humoral immune responses in different age subgroups.** Serum IgG anti-RBD of the SARS-CoV-2 were determined at several times post Gam-COVID-Vac in individuals classified into the following age subgroups: 70-74, 75-79, 80-84 and 85-96 years old. Horizontal red lines represent the median.

As much higher risk of developing severe COVID-19 in male patients may be a result of an enhanced innate immunity and/or an impaired T cell activation compared with females [20], we decided to analyzed if the Gam-COVID-Vac had been able to elicited a robust immune response in both male and female. In the elderly population analyzed in our study, no significant differences were observed between genders in the specific humoral immune response to Gam-COVID-Vac. Similar median IgG anti-RBD titers were observed in female and male volunteers after receiving the Gam-COVID-Vac across all time-points studied (14, 28, 90 and 180 dpv) except at 60 dpv, where the male group exhibited a statistically significant reduced median titer (Figure 3). This difference, however, disappeared at 90 and 180 dpv. In order to determine if humoral immune kinetics elicited by Gam-COVID-Vac varied according with age specifically within our elderly cohort, we performed a deeper analysis by dividing the volunteers into specific subgroups, 70–74; 75–79; 80–84 and 85–96 years old. We found that although antibody response curves were similar, peaking at 28 dpv (Figure 4), the highest antibodies titers were observed in the youngest subclass (70–74 years) when compared the other three older subgroups analyzed, and this was not due to a specific enrichment of individuals with basal anti-RBD titers by previous SARS-CoV-2 infection.

## DISCUSSION

Aging is an important risk factor for severe COVID-19 disease including hospitalization, intensive care unit admission, and death. The mortality rates of up to 54% have been reported for COVID-19 patients above the age of 80 [21]. These observations indicate the necessity to shed light on the outcome, protective measures, and the perspective of this disease on the geriatric population [2, 17]. Even though elderly people are likely to be among the first to be vaccinated [22], these patient groups are usually excluded from clinical trials and there is scarce published data on the efficacy of vaccines in this group [23]. The present study provides new data regarding the persistence of antibody responses over time to the two doses of the Gam-COVID-Vac vaccine in an elderly group of volunteers in San Miguel de Tucumán, Argentina. A limitation of this study was the progressive drop-out observed, which can be explained by the characteristics of the elderly population, who have difficulties to assist to sampling, and by the irruption of the second wave of COVID-19.

At 28 dpv, the seroconversion rate raised up to 91% (Figure 1B). We consider this level of seroconversion in the elderly population as more than adequate, considering that immunosenescence is a pending challenge for immunologists, as is achieving an efficient stimulation to new antigens in elderly people.

Elderly volunteers with detectable anti-RBD antibodies at baseline, developed higher antibody titers within 14 days of vaccination, similar to what has been shown before in younger individuals [19, 24–27]. These results emphasize the importance of administering, in elderly individuals, at least two doses of Gam-COVID-Vac in order to achieve a robust immune response, especially in individuals without a previous SARS-CoV-2 infection.

No significant differences were observed in the IgG anti-RBD antibodies in male and female volunteers, except at 60 dpv where anti-RBD titers were more persistent in elderly females, and only dropped at 90 dpv (*p* < 0.0001). This increased persistence of SARS-CoV-2 specific antibodies in females, also reported by Søgaard OS [28], can be explained by the presence of more active B and T cells in older females, resulting in the generation of long-term responses to antigens [29]. Although males have been shown in some cases to exhibit reduced humoral immunity after vaccination for, or infection with, SARS-CoV-2 [30, 31], due to the crucial role of cellular immunity in defense against this virus, a decrease in specific antibodies should be carefully studied and not immediately assumed as a reduction in protection towards severe COVID-19.

Previous studies have shown that neutralizing antibody responses can be induced in older adults by different vaccine platforms, including mRNA [32, 33] adenovirus vectors [34, 35] and inactivated virus [36]. More recently, Wu Z [37] analyzed the safety, tolerability, and immunogenicity of an inactivated SARS-CoV-2 vaccine (CoronaVac) in healthy adults aged 60 years and older in a randomized, double-blind, placebo-controlled, phase 1/2 clinical trial, and demonstrated that neutralizing antibody responses to live SARS-CoV-2 are not reduced in this population [37].

When anti-RBD titers (measured with the same assay) elicited in elderly adults were compared to those elicited in younger healthcare workers (18 to 60 years old) that had also received the complete Gam-COVID-Vac scheme [23], we found higher antibodies levels in the latter group. This difference in antibodies titers between both populations could be due to age-related immunosenescence, and the profound reduction in B cell populations and associated antibody production [38, 39].

There are few studies that have compared the immune responses to Gam-COVID-Vac with other available vaccines specifically in elderly individuals. For example, Jeewandara and colleagues investigated the immunogenicity of a single vaccine dose of Gam-COVID-Vac in Sri Lanka and found percentages of seroconversion significantly lower (81.8%) in those over 60 years and significantly lower than previously seen with AZD1222. Similar titers of antibodies were observed to the RBD of WT, B.1.1.7 and B.1.617.2 compared to AZD1222, while the levels for B.1.351 were significantly higher for Gam-COVID-Vac [40]. In Argentina, a retrospective study carried by Gonzalez et al. (2021) in individuals aged 60–79 of the Province of Buenos Aires, Argentina, showed that Gam-COVID-Vac prevents 78.6% of laboratory-confirmed SARS-CoV-2 infections, 87.6% of hospitalizations and 84.8% of deaths at 83 days after vaccination [41]. Petrovic et al., provided evidence about the vaccine effectiveness in subjects aged ≥60 years fully vaccinated, reaching values as high as 86.9% for BBIBP-CorV (Sinopharm vaccine), 95% for Gam-COVID-Vac and 99% for BNT162b2 [42]. In a different analysis stratified by age, the protection elicited after complete vaccination with Vaxzevria (a viral-vector vaccine) and CoronaVac (inactivated virus vaccine) was compared [43]. While Vaxzevria induced a vaccine efficacy (VE) around 90% for aged up to 89 years, CoronaVac-VE reached around 75% protection. Interestingly, the authors showed that in the group up to 90 years old, reduction in vaccine efficacy is most likely associated with ageing regardless of the vaccine administrated. In the context of new SARS-CoV-2 variants of concern, and the fact that the elderly individuals represent a vulnerable population, the precise time point in which antibodies begin to decrease can provide valuable information to understand the whole immune response elicited against SARS-CoV-2.

Diminished immune responses and higher mortality rates to SARS-CoV-2 in older individuals may also be due to increased comorbidities, such as obesity, hypertension, or diabetes, commonly associated with aging [44, 45]. It has been reported that low levels of antibody production are associated with obesity, where elevated levels of circulating leptin induce a diminished immune response, together with high levels of pro-inflammatory cytokines [46–48]. Other authors reported a correlation between obesity and defects in immune surveillance, suggesting that this condition represents a potent modifier of immunosenescence [49]. In individuals with diabetes, another comorbidity associated with age, hyperglycemia causes dysfunction of the immune response, and a subsequent failure to control the spread of invading pathogens [50].

Even though the exact role of senescence in COVID-19 is not fully understood, vaccines have proven an unquestionable strategy against infectious disease. The present study offers new data regarding the humoral immune responses elicited to Gam-COVID-Vac in an elderly population. Is it important to mention that the immune response elicited upon the two doses of Gam-COVID-Vac protected this elderly group to a SARS-COV-2, at least up to 180 dpv, as only one male volunteer (72 years old) got infected in the time of the study, and did not require hospitalization (Table 1). Nevertheless, the drastic drop in the specific titers observed after 90 dpv reveal the importance of boosting the immune system, before that time, with a third vaccine dose, as shown by others for other platforms and younger age groups [51, 52]. We believe that our study can contribute independent knowledge on the immune response in an immunosenescence context, such those in elderly individuals, to a vaccine platform administered to tens of millions worldwide but has been scarcely studied. In addition, there is an underrepresentation of elderly individuals in clinical trials on COVID-19 vaccines, even though they are the most affected by this disease [53].

As the aging population is increasing globally, especially in developed countries, vaccine efforts must consider age-related issues in order to ensure effectiveness. Ongoing studies will provide data regarding the best strategy to strengthen and prolong the protective immune response against COVID-19 in the elder population, challenging the immunosenescence process, to ameliorate the severity of the disease and avoid the SARS-CoV-2 infection.

## MATERIALS AND METHODS

### Population study

This study was approved by the Tucumán Public Health System Research Committee: https://msptucuman.gov.ar/wordpress/wp-content/uploads/2021/03/Expediente-N3929-410-P2020-Seroconversin-post-vacunacin-para-SARS-CoV-2-immune-response-after-a-single-vaccine-dose.pdf.

The Ethics Committee of the Ministry of Health of the Province of Tucumán evaluated and approved the protocol of the present study (Ex. No 3929–410-P-2020), https://msptucuman.gov.ar/wordpress/wp-content/uploads/2021/03/03.Serocoversion-DICTAMEN-19-1-2021.pdf, following the Declaration of Helsinki.

Volunteers between 70 to 96 years old to be vaccinated with Gam-COVID-Vac between December 2020 and February 2021 in different vaccination nodes were invited to participate in the study. The inclusion criteria were: people from 70 to 90 years old, without COVID-19 symptoms at the time enrolment, and enlisted to receive the complete two-dose Gam-COVID-Vac vaccination scheme. and had signed the Informed Consent to participate in this study. The exclusion criteria were individuals with current COVID-19 symptoms (dry cough, fever, dyspnea) or current COVID-19 diagnosis at the time of enrollment.

All enrolled volunteers were properly informed regarding their choice to participate in the study and provided a signed informed consent (forms can be found at https://msptucuman.gov.ar/wordpress/wp-content/uploads/2021/03/FCISeroconversionRCSfinal.pdf). Personal data from all volunteers were encrypted.

Anti-RBD titers of convalescent individuals (CP) acquired with the same ELISA platform were obtained from previous studies [17, 18]. This population was defined as individuals diagnosed with both RT-PCR and serology with both anti-RBD IgG ELISA and anti-N CMIA) who provided serum samples 28–42 days-post diagnosis with SARS-CoV-2 infection and with a median age of 36.9 ± 13.6.

### Anti-RBD ELISA assay

Serum RBD-specific IgG antibody titers were determined by an “*in house* ELISA” as previously described [17, 18]. Briefly, 0.1 μg of recombinant RBD from SARS-CoV-2 was immobilized in each well of a 96-well flat polystyrene bottom plates (High Binding, Half-Area, Greiner #675,061). Blocking was performed with 10% Fetal Bovine Serum in PBS 10 mM pH7.4 during 1 h at 37°C. Sera (1/100) were assayed at serial dilutions in 10% Fetal Bovine Serum in PBS and incubated for 1 h at 37°C. Peroxidase-conjugated immunoglobulins to human IgG (whole molecule) (Sigma A8667), diluted 1/35,000, were used as the secondary antibody. Plates were developed by adding TMB (3,3′,5,5′–Tetramethylbenzidine; BD OptEIAtm), and the reaction was terminated using 4N H_2_SO_4_. Optical density (OD) was read by an ELISA reader (TECAN Spark) at 450 nm. Titers were calculated as the dilution in which the optical density obtained was equal to the cutoff.

### Statistical analysis

Statistical analyses were performed using non-parametric tests in Prism 8.0 software (GraphPad, San Diego, CA, USA). The post-tests applied in each trial are indicated in the figure legends. Normality of the data distribution was performed with the Shapiro –Wilk test. Data that did not shown a normal distribution were depicted as medians. Differences among the distribution between two groups were analyzed with the Mann-Whitney *U* test. The median anti-RBD titers of all groups were compared using Kruskal-Wallis with a correction for multiple comparisons by controlling the false discovery rate using the two-stage linear step-up procedure of Benjamini, Krieger and Yekutieli. Statistical significances are indicated in the figures by asterisks as follows ^*^p < 0.05, ^**^p < 0.01, ^***^p < 0.001. In all cases, a *p*-value of less than 0.05 was assumed as statistically significant.

## Supplementary Materials

Supplementary Figure 1
