# Supplementary material for: Humoral immunoresponse elicited against an adenoviral-based SARS-CoV-2 coronavirus vaccine in elderly patients
Source: Aging (Albany NY). 2022 Sep 21;14(18):7193–205. doi: 10.18632/aging.204299 (PMC9550251; doi:10.18632/aging.204299)
Supplement: Supplementary Figure 1 [file aging-14-204299-s001.pdf]

SUPPLEMENTARY FIGURE

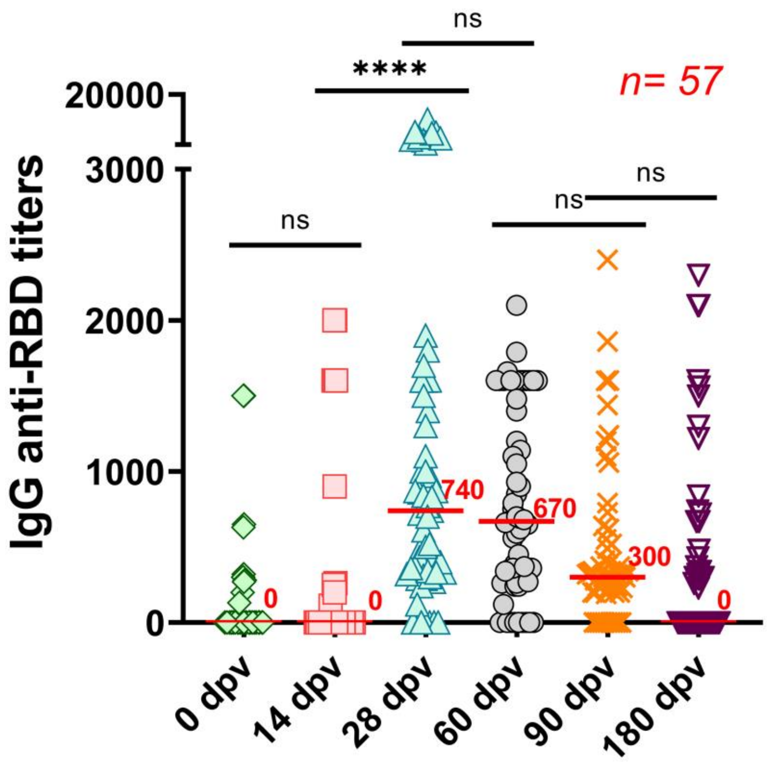

Supplementary Figure 1. Longitudinal analysis of humoral immune response against SARS-Cov-2 in 57 elderly individuals. Ig anti-RBD antibodies were determined in serum samples taken at 0, 14, 28, 60, 90 and 180 days' post-vaccination (dpv) by an "*in house*" ELISA platform.
